# Predictive Factors of Nonmalignant Pathological Diagnosis and Final Diagnosis of Ultrasound-Guided Cutting Biopsy for Peripheral Pulmonary Diseases

**DOI:** 10.1155/2023/5815755

**Published:** 2023-06-09

**Authors:** Qing Li, Li Zhang, Xinhong Liao, Yanfen Zhong, Zhixian Li

**Affiliations:** ^1^Department of Diagnostic Ultrasound, The Affiliated Hospital of Youjiang Medical University for Nationalities, Baise 533000, Guangxi, China; ^2^Department of Diagnostic Ultrasound, First Affiliated Hospital of Guangxi Medical University, Nanning 530021, Guangxi, China

## Abstract

This study aimed to explore the predictive factors of nonmalignant pathological diagnosis and final diagnosis of ultrasound-guided cutting biopsy for peripheral pulmonary diseases. A total of 470 patients with peripheral lung disease diagnosed as nonmalignant by ultrasound-guided cutting biopsy in the First Affiliated Hospital of Guangxi Medical University from January 2017 to May 2020 were included. Ultrasound biopsy was performed to determine the correctness of pathological diagnosis. Independent risk factors of malignant tumor were predicted by multivariate logistic regression analysis. Pathological biopsy results showed that 162 (34.47%) of the 470 biopsy data were specifically benign, and 308 (65.53%; malignant lesions: 25.3%, benign lesions: 74.7%) were nondiagnostic findings. The final diagnoses were benign in 387 cases and malignant in 83 cases. In the nondiagnostic biopsy malignant risk prediction analysis, lesion size (OR = 1.025, *P* = 0.005), partial solid lesions (OR = 2.321, *P* = 0.035), insufficiency (OR = 6.837, *P* < 0.001), and presence of typical cells (OR = 34.421, *P* = 0.001) are the final important independent risk factors for malignant tumors. In addition, 30.1% (25/83) of patients with nonmalignant lesions who were finally diagnosed with malignant tumors underwent repeated biopsy, and 92.0% (23/25) were diagnosed during the second repeated biopsy. 59.0% (49/83) received additional invasive examination. Nondiagnostic biopsy predictors of malignant risk include lesion size, partial solid lesions, insufficiency, and presence of atypical cells. When a nonmalignant result is obtained for the first time, the size of the lesion, whether the lesion is subsolid, and the type of pathology obtained should be reviewed.

## 1. Introduction

More than 2 million new cases of lung cancer and about 1.79 million patients who died from lung cancer were reported in 2020, which remained the leading cause of death among patients with cancer [1]. An increasing number of patients have been found to meet the screening criteria for lung cancer using CT scans. Therefore, the incidence and detection rate of peripheral pulmonary diseases have gradually increased in recent years [2, 3]. Percutaneous lung biopsy guided by imaging technology is a useful and well-known method. Peripheral pulmonary diseases are located in the segment and below the bronchus and close to the chest wall, which provides the feasibility of ultrasound. Ultrasound can provide precise positioning and visual detection. It is a nonradiation, fast, economical, and effective guidance method. Ultrasound-guided puncture biopsy of peripheral pulmonary diseases has an acceptable diagnostic rate, and the complication rate is lower than that of CT-guided biopsy [4–7]. Therefore, ultrasound should be recommended because peripheral pulmonary diseases can be observed through the use of ultrasound. Tissue biopsy is the gold standard for diagnosing benign and malignant lung nodules or tumors [8]. The diagnosis of lung malignant tumors, benign-specific diagnosis, and the presence of positive biopsy cultures by percutaneous lung biopsy are considered as true reflections of the disease [9–11]. However, when inconclusive diagnoses such as nondiagnostic results or insufficient specimens are obtained, the reliability of the biopsy using this technology is low, which puts clinicians into a dilemma [12, 13]. Based on a multicenter study in recent years, the negative predictive value of CT-guided lung biopsy for the diagnosis of malignant diseases is only 51% [14]. Therefore, accurately diagnosing the pathological characteristics of these nodules or masses and distinguishing malignant and benign lesions are necessary for treatment planning.

Although some relevant studies have been published, the number of studies that analyze the initial pathological report of percutaneous lung biopsy that yielded a nonmalignant diagnosis and the number of cases included in peripheral pulmonary diseases are limited [13–15], and they primarily focus on CT as an image-guided method or fine needle aspiration method [15–17]. In the vast majority of research reports, no research has focused on the analysis of large sample sizes of nonmalignant lesions in the first biopsy of nonmalignant lesions under the guidance of ultrasound. Therefore, this study aims to analyze the final diagnosis of nonmalignant lesions in the initial biopsy and the predictive factors of benign and malignant nondiagnostic lesions based on a larger sample of data.

## 2. Materials and Methods

### 2.1. Study Objects

A total of 957 percutaneous lung biopsy data of patients diagnosed with peripheral lung disease and accompanied by ultrasound-guided percutaneous lung biopsy in the First Affiliated Hospital of Guangxi Medical University from January 2017 to May 2020 were selected, and 487 biopsy data with malignant diagnosis were excluded. The inclusion criteria were as follows: (1) peripulmonary space occupying lesions confirmed by CT; (2) have indications for lung biopsy and the family members sign the informed consent of biopsy; and (3) ultrasonography can clearly show the focus. The exclusion criteria were as follows: (1) patients with contraindications of lung biopsy (coagulation dysfunction; severe cardiopulmonary insufficiency such as severe pulmonary hypertension; anatomic or functional isolation of the lung; evident infectious lesions along the puncture path, bullosa, chronic obstructive pulmonary disease, emphysema, and pulmonary fibrosis; mechanical ventilation (ventilator); radiographic consideration of pneumoechinococcosis); (2) those who received ultrasound-guided percutaneous biopsy without pathological examination; and (3) malignant disease diagnosed by puncture biopsy. The general information of the patient was recorded. This retrospective single-center study was approved by the ethics committee of the First Affiliated Hospital of Guangxi Medical University (approval number: 2021[KY-E-199]). Given the retrospective nature of this study, the requirement to sign informed consent was exempted.

### 2.2. Puncture and Biopsy

Bard biopsy needle (16 G and 18 G types) was soaked in 75% ethanol solution and covered with a disposable sterile plastic film for 10 min to ensure sterility. GE logiq E9 or Siemens Acuson S2000 conventional ultrasonic diagnostic instrument was adopted, and the probe frequency was 2.5–4.0 MHz or 3.55.5 mhz. Based on the specific location and size of the lesion, blood supply, the adjacent relationship of the lesion with the surrounding tissues displayed on the ultrasound image, patient's body position, and the best puncture path were determined. Routine disinfection, lying of surgical towel, and administration of local anesthesia with 1-2 mL of 2% lidocaine were described. Avoiding the heart and large blood vessels, the needle tip of the Bard adjustable biopsy gun was inserted into the target lesion in accordance with the predesigned puncture path. When the needle path was clearly displayed, the patient was required to hold his breath, trigger the shooting, and withdraw the needle. Afterward, the sample was stored in 4% formaldehyde solution for pathological examination. The occurrence of complications such as hemoptysis, pneumothorax, and pleural reaction was observed.

### 2.3. Definition of Initial Diagnosis

Malignant diagnosis refers to malignant tumors, malignant cells, and pulmonary sequestration. Nonmalignant diagnosis is characterized as follows [13, 16, 18]:

1. Diagnostic group: specific benign: hamartoma, tuberculosis, fungus, pulmonary inflammatory pseudotumor, etc.
2. Nondiagnostic group: nonspecific benign: organized pneumonia, suppurative inflammation, granulomatous inflammation, and inflammatory or fibrous exudation and other inflammatory processes described but not diagnosed. Inconclusive results: insufficient specimens: the presence of normal respiratory system components, blood, necrosis, insufficient specimens, and unexplainable conditions. Dysplasia: presence of atypical cells.

If the patient performs two or three biopsies on the same target lesion, these repeated biopsies are counted as independent events.

### 2.4. Final Diagnosis

The final diagnosis was performed in combination with patient's medical history and subsequent treatment examination results [16, 19, 20]. The final diagnostic criteria were as follows: (1) if all diagnostic results are consistent, the clear diagnosis is confirmed; (2) the results of surgical pathology report and biopsy report are contradictory, and the surgical case report shall prevail; (3) when the pathological results include malignancy, the malignant diagnosis is used; and (4) the patients were followed up for at least half a year. In the absence of chemotherapy, radiotherapy, and anticancer drug treatment, the CT results showed that the diameter of the lesions decreased or remained unchanged, and they were diagnosed as benign lesions. In the presence of evident metastasis or progression of malignancy during follow-up CT, the diagnosis is considered as malignant.

### 2.5. Statistical Analysis

Using SPSS 25.0 for statistical analysis of data, continuous variables were expressed as mean ± standard deviation (SD), and two independent sample *t*-test was used for comparison between the two groups. Categorical variables were expressed by frequency (percentage), and the chi-squared test was used for statistical analysis. Multivariate logistic regression analysis was used to test the influencing factors of malignant lesions. *P* < 0.05 was considered statistically significant.

## 3. Results

### 3.1. General Information

**Figure 1:**
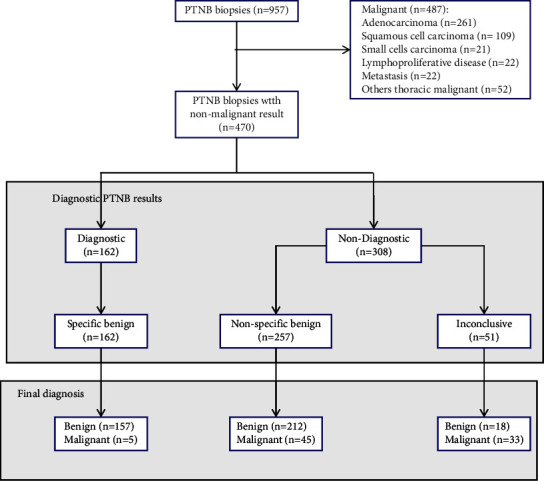
Flow diagram of patients and outcomes of initial percutaneous transthoracic needle biopsies (PTNBs) of the peripheral pulmonary.

Figure 1 illustrates the flow chart and diagnostic results of patient screening for initial PTNBs. Also, the baseline characteristics are shown in Table 1. A total of 470 patients diagnosed with nonmalignant lesions underwent ultrasound-guided puncture biopsies. The average age of the patients was 53.2 ± 14.0 years (age range 15–80 years). Out of the included patients, 72.8% (342/470) were male, and 27.2% (128/470) were female. The average size of the lesion was approximately 31.2 ± 16.2 mm (range of approximately 8–103 mm).

Among the 470 nonmalignant diagnostic biopsies, the distribution of initial pathological diagnosis and final diagnosis is shown in Table 2. Among the 470 patients, 34.5% (162/470) had specific benign diagnostic results; 54.7% (257/470) had a nonspecific benign diagnostic result, and 10.9% (51/470) had inconclusive results. Among the 162 cases of specific benign diagnostic biopsies, 3.1% (5/162) were diagnosed as malignant, and 96.9% (157/162) were diagnosed as benign lesions. In addition, among the 257 cases of nonspecific benign diagnostic biopsies, 17.5% (45/257) of the final diagnosis was malignant, and 82.5% (212/257) was finally diagnosed as benign lesions. Among the 51 cases of inconclusive lesion diagnosis, 90.9% (10/11) of 11 cases of atypical cell diagnosis were finally diagnosed as malignant, and 9.1% (1/11) were finally diagnosed as benign lesions. Out of the 40 cases with insufficient tissue diagnosis, 57.5% (23/40) was finally diagnosed as malignant, and 42.5% (17/40) was finally diagnosed as benign. Among the 470 included cases of nonmalignant diagnosis, the final diagnosis of benign and malignant lesions accounted for 82.3% (387/470) and 17.7% (83/470), respectively (Table 2).

### 3.2. Multivariate Logistic Regression Analysis of Nondiagnostic Biopsy

Among the 308 nondiagnostic biopsies, the final diagnosis of benign and malignant was 74.7% (230/308) and 25.3% (78/308), respectively. The demographic data and lesion characteristics were compared between the benign group and the malignant group of 308 nondiagnostic biopsies (Table 3). Malignancy mostly occurred in larger masses (>15 mm, *P*=0.001), the right lung (*P* < 0.001), and the middle or lower lobe of the lung (*P* < 0.001), and the prone position was more adopted during puncture (*P*=0.01). In addition, atypical cells and insufficient diagnostic tissue had a higher risk of final malignancy than nonspecific inflammatory lesions (*P*=0.001). No statistically significant differences in gender, age, nature of the lesion, cutting needle type, puncture angle, and puncture times were observed.

Furthermore, the size of the lesion, lesion location 1 (left lung, right lung), lesion location 2 (upper, middle, or lower lobe), and pathological characteristics (nonspecific benign lesions, atypical hyperplasia, and insufficient tissue) were used as a factor regression variable. A multivariate regression model was established, and the results indicated that the lesion size (OR, 1.025; 95% CI: 1.007–1.043; *P*=0.005), subsolid lung lesion (OR, 2.321; 95% CI: 1.057–4.712; *P*=0.035), insufficient tissue (OR, 6.837; 95% CI: 3.210–14.563, *P* < 0.001), and the presence of atypical cells (OR, 34.421; 95% CI: 3.959–299.263; *P*=0.001) were the final important independent risk factors for malignant tumors (Table 4).

### 3.3. Final Malignant Diagnosis and Initial Diagnosis of 83 Nonmalignant Lesion Cases

**Figure 2:**
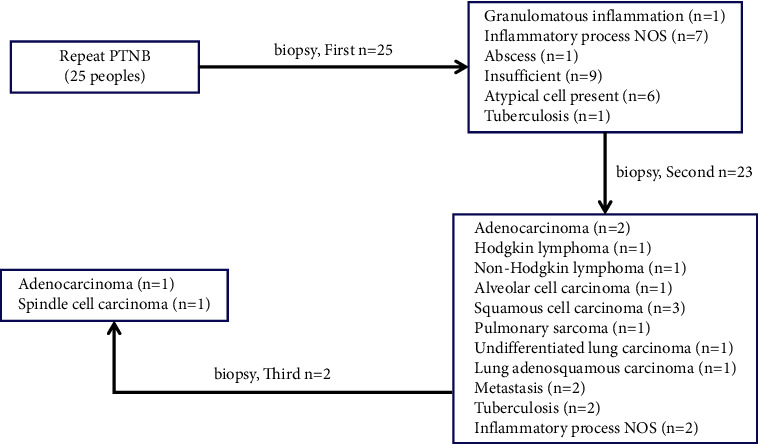
Analysis of data from repeated biopsies (25 patients).

The results of 83 cases of initial diagnosis of nonmalignant lesions and final diagnosis of malignancy were further evaluated, out of which 30.1% (25/83) received repeated biopsy and 92.0% (23/25) received second repeated biopsy to confirm the diagnosis. In addition, 8% (2/25) underwent a third puncture biopsy, which was finally diagnosed as malignant. Additional invasive examinations were received by 59.0% (49/83), and 10.8% (9/83) confirmed the diagnosis of malignancy through clinical follow-up (Figure 2 and Table 5).

## 4. Discussion

In this study, the predictive factors of nondiagnostic pathological results and potential malignant tumors, the size of the lesion, the type of the lesion, the presence of atypical cells, and the insufficiency of tissue were evaluated through analysis of baseline data, image characteristics, operation-related techniques, and pathological types. Each pathological type of nondiagnostic lesions has a different rate of malignancy. The malignancy rate of atypical cells is as high as 90.0% (10/11), followed by 57.5% (23/40) of insufficient tissue, and that of nonspecific benign lesions is 17.5% (45/257). Through the follow-up of the nonmalignant results of the initial puncture biopsy, 30.1% (25/83) had repeated biopsies, 59.0% (49/83) received additional invasive examinations, and 10.8% (9/83) confirmed malignancy through clinical follow-up diagnosis.

In a retrospective analysis of 470 cases of ultrasound-guided cutting biopsy of peripheral pulmonary diseases for the first pathological diagnosis of nonmalignant lesions during 3.5 years, the final diagnosis of benign and malignant lesions accounted for 82.3% (387/470) and 17.7% (83/470), respectively. In some of the published studies on lung lesion biopsy guided by imaging technology, the incidence of initial nondiagnostic biopsy of malignancy accounted for 16.4%–50% [10, 14, 17, 21]. Therefore, our research results are consistent with the literature reports of previous studies. In a study of transthoracic CT-guided coaxial puncture aspiration biopsy, Rui et al. finally determined 141 malignant cases (16.4%) and 720 benign cases (83.6%) [17]. Similar to our results, the 141 patients (141/861, 16.4%) who were finally diagnosed with malignancy in the study of Rui et al. were all from the “unspecified infection,” “inflammatory disease,” or “indeterministic” group [17]. Among the 83 patients who were finally diagnosed with malignancy in this study, five had specific benign lesions such as tuberculosis and inflammatory pseudotumors at the first diagnosis. In a study of the predictive factors and final diagnosis of nondiagnostic results of CT-guided percutaneous lung biopsy, Tongbai et al. [15] found that 36% of nondiagnostic cases were ultimately diagnosed with malignancy. In this study, only 17.7% of nondiagnostic cases had a malignant diagnosis, which was significantly lower than that reported by Tongbai et al. This difference may be due to the broader definition of nondiagnostic biopsy in this study. Tongbai et al. attributed pathological findings such as organized pneumonia and granulomatous inflammation to specific benign diagnosis. In our study, organized pneumonia, granulomatous inflammation, and abscesses, which have inconclusive specificity, are classified as nonspecific benign lesions.

Among the 470 patients included in this study, the proportion of specific benign was 34.5% (162/470), which was higher than that (21%) reported by Quint et al. in a study on the value of CT-guided coaxial puncture biopsy with negative results. This study is similar to Quint et al.'s study [22] in determining the diagnostic criteria for specific benign lesions probably because the specific benign ratio of tuberculosis in the pathological diagnosis of puncture biopsy in this study is as high as 63.0% (102/162). Based on literature, our country is one of the countries with the heaviest tuberculosis burden worldwide. Tuberculosis has a high incidence rate in our country [23], whereas Quint et al. conducted research in the United States, where the number of patients with tuberculosis is limited. However, compared with the specific benign diagnostic rate of 77% (17/22) reported by Satoh et al. [24], the reason why the diagnostic rate of specific benign lesions in our study is lower than that in this study remains unclear probably because of the differences in countries, ethnic groups, or manifestations of the disease.

Among the 308 nondiagnostic biopsies in this study, the percentage of nonspecific benign lesions, atypical cells, and insufficient pathological report diagnosis was about 83.4% (257/308), 3.6% (11/308), and 13% (40/308). JOO-WON et al. reported on the clinical significance of nondiagnostic pathological results of percutaneous lung biopsy: a tertiary hospital without on-site cytopathologists was reported in 103 cases of nondiagnostic biopsy patients with nonspecific inflammation. The percentages of atypical cells and insufficient specimens were 50.5% (52/103), 15.5% (16/103), and 40% (35/103), respectively. The proportion of nonspecific inflammation in our study is significantly high, whereas the proportion of atypical cells and pathologically reported insufficient tissue is low probably because a relatively large cutting needle of 16 G or 18 G was used in this study, which improves the output and accuracy of the diagnosis. In a multicenter study on nondiagnostic pathology, the risk assessment of malignant tumors by percutaneous lung biopsy conducted by Lee et al. [16] found that the percentage of nonspecific inflammation, atypical cells, and insufficient specimens was 61.5%, 21.5%, and 17.0%, respectively. Our nonspecific inflammation ratio is high; the insufficient tissue is low; and the presence of typical cells is significantly low. This difference in results might be due to the fact that the study conducted by Lee et al. [16] was guided by CT, radiation, ultrasound, and other imaging techniques. In the selection of puncture needle type, fine needle aspiration and cutting needle cutting were used alone or in combination. However, this study is purely ultrasound-guided puncture biopsy of peripheral pulmonary diseases, and it uses a large 16 G or 18 G cutting needle, which is sufficient for obtaining tissue samples. Most of the tissue specimens obtained can be examined by immunohistochemistry, thereby improving the diagnosis. With regard to the proportion of final malignancy in this study, nonspecific benign lesions, insufficient tissue, and atypical cells accounted for 17.5% (45/257), 57.5% (23/40), and 90.9% (10/11), respectively. This finding is consistent with the results of previous studies, with 21.3%–37.2% and 46.6%–50% for nonspecific benign tumors and insufficient specimens, respectively, in the final diagnosis of malignancy under the guidance of imaging technology [16, 17, 22]. We reported that the proportion of the final malignant diagnosis of atypical cells is higher than that reported by other studies (62.5% or 64.3%, 10/14) [13, 15], which is similar to the results of Lee et al. [16] in a large multicenter sample study. In this study, by observing the risk of final malignancy of pathological types in nonmalignant diagnosis, compared with nonspecific benign lesions and insufficient specimen pathological report diagnosis, atypical cells likely have malignancy. In addition, when the puncture biopsy shows nonspecific benign lesions or insufficient tissue, the possibility of malignancy cannot be completely ruled out.

In the univariate analysis of this study, lesion location 1 (right lung, left lung), location 2 (upper lobe, middle or lower lobe), and patient's position (supine, lateral, and prone) are considered as predictors. Malignancy is common in the right lung as well as middle and lower lobe, and the prone position is commonly used during puncture. After multivariate analysis, *P* values of the abovementioned variables are all >0.05. In multivariate analysis, the predictive factors of the nondiagnostic outcome of malignancy include the size of the lesion, the type of the lesion, the presence of atypical cells, and the insufficient tissue for diagnosis. The variable related to malignancy in this study is the size of the lesion. This view is consistent with the findings of Rui et al. [17]. Therefore, in nondiagnostic lesions, the larger the lesion, the higher the risk of malignancy. Some literature reports that the larger the lesion, the higher the incidence of false negatives [25–27]. Gelbman et al. [9] also pointed out that larger lesions are more prone to false negatives in their radiological and clinical characteristic studies on the false negative results of CT-guided lung nodule biopsy. In this study, after multivariate analysis, subsolid nodules are more likely to be malignant than solid nodules because of various factors, which is consistent with the results of previous studies [28, 29]. However, Yun et al. [30] compared 354 patients with lung biopsy guided by CT and analyzed the diagnostic rate, biopsy-related factors, and complications of solid and subsolid lesions, and the results showed that nondiagnostic biopsy had no statistical difference between solid and subsolid lesions. Lee et al. [16] retrospectively analyzed 2590 lung biopsy cases and classified them into three categories: nonspecific benign lesions, atypical cells, and insufficient specimens. The results show that atypical cells that are suspected of being malignant are more likely to be diagnosed more malignant than atypical cells that are inconclusive. In nonspecific benign lesions, granulomatous inflammation, abscesses, and organized pneumonia are independent factors for eliminating malignancy. In a multivariate analysis of 122 cases of benign and malignant nondiagnostic lesions, Tongbai et al. [15] proposed that nondiagnostic biopsies with a history of malignancy or pathologically atypical cells are more likely to be malignant. In this study, the presence of atypical cells is an important independent risk factor for the final diagnosis of nondiagnostic lesions as malignancy, which is consistent with the abovementioned literature reports. During tissue sampling, due to various reasons such as lesion size and location, tissue sampling was insufficient and sample size was small, so it was possible that the lesion site could not be obtained, resulting in false negative results. When there is a benign diagnosis due to insufficient sample size, the possibility of malignancy cannot be ruled out, and we should conduct verification again if conditions permit.

In our study, 30% (25/83) of 83 patients who were initially diagnosed with nonmalignant lesions and finally diagnosed as malignancy underwent repeated biopsy, 59.0% (49/83) underwent additional invasive examination, 10.8% (9/83) confirmed the diagnosis of malignancy through clinical follow-up radiology. 25 cases underwent the second repeat biopsy, of which 23 (92%, 23/25) finally confirmed the diagnosis, and 2 cases confirmed the malignant diagnosis after the third repeat biopsy. It can be seen from this study that a higher diagnostic rate can be obtained through repeated biopsy. Previous research reports have also confirmed this view [14–16, 25]. Montaudon et al. [26] found that the same target had a negative predictive value of 100% in the second biopsy. In a study of 950 transthoracic CT-guided biopsy patients with nonmalignant lesions found for the first time, Rui et al. [17] pointed out that 47.6% of patients underwent additional invasive tests to confirm the final diagnosis. In this study, 59.0% (49/83) received additional invasive tests to confirm the diagnosis of malignancy. Therefore, for the biopsy results of the nondiagnostic lesions, additional diagnostic tests should be performed when necessary. Savage et al. [21] also put forward this point of view.

This study has certain limitations: first, it is a retrospective analysis study, some data lacking is excluded, and there is a certain bias. Second, this study failed to analyze the patient's malignant tumor history, smoking history, family history of lung cancer, and complications of puncture biopsy. Finally, since most puncture biopsies are used to clearly diagnose lung lesions that are suspected of being malignant, in further research, the accuracy and diagnosis failure of malignant tumors will be studied and analyzed.

## 5. Conclusion

The diagnostic rate of ultrasound-guided cutting biopsy for peripheral pulmonary diseases is acceptable, which has certain application value in clinic. The predictors of benign and malignant nondiagnostic biopsy include the presence of atypical cells, the insufficiency of diagnostic tissues, and the type of the lesion and the size of the lesion. Therefore, when a nonmalignant result is obtained, the lesion should be reviewed to determine whether there are any factors, including the size of the lesion, the type of the lesion, and the type of pathology obtained. If necessary, a repeated biopsy or additional invasive examination should be performed. Such additional evaluation and careful monitoring can help doctors further identify nonmalignant peripheral pulmonary diseases to confirm the final diagnosis.

## Figures and Tables

**Table 1:** Baseline characteristics of the study population (*N* = 470).

Characteristics	*n*/mean ± SD	(%)
*Age (years)*		
Range	15–80	
Mean ± SD	53.2 ± 14.0	

*Gender*		
Male	342	72.8
Female	128	27.2

*Smoking history*		
Smoker	241	51.2
Nonsmoker	229	48.7

*Lesion location 1*		
Left lung	212	45.1
Right lung	258	54.8

*Lesion location 2*		
Upper lobe	180	38.2
Middle and lower lobe	290	61.2

*Lesion size (mm)*		
Range	8–103	
Mean ± SD	31.2 ± 16.2	

*Lesion type*		
Solid	348	74.0
Subsolid	122	25.9

*Patient position*		
Supine	84	17.8
Lateral	152	32.3
Prone	234	49.7

*Needle type (gage)*		
16	41	8.7
18	429	91.2

*No. of tissue samplings*		
≤2	140	29.7
= 3	256	54.4
>3	74	15.7

*Puncture angle*		
0°	106	22.5
15°	323	68.7
30°	16	3.4
Multiangle	25	5.3

**Table 2:** Distribution of nonmalignant diagnoses for initial biopsies and final diagnoses.

Initial pathological diagnosis		Final	
Nonmalignant diagnosis	Category of nonmalignancy	Benign	Malignant

Specific benign (162, 34.5%)	Tuberculosis (102)	157 (96.9%)	5 (3.1%)
	Pulmonary inflammatory pseudotumor (34)		
	Solitary fibrous tumor (1)		
	Neurilemmoma (3)		
	Pulmonary cryptococcosis (19)		
	Pulmonary aspergillosis (2)		
	Pulmonary sclerosing hemangioma (1)		

Nonspecific benign (257, 54.7%)	Organized pneumonia (58)	212 (82.5%)	45 (17.5%)
	Interstitial lung disease (16)		
	Granulomatous inflammation (19)		
	Abscess (25)		
	Fibrosis process (12)		
	Inflammatory process NOS (127)		

Inconclusive result (51, 10.9%)	Atypical cell present (11)	1 (9.1%)	10 (90.9%)
	Insufficient (40)	17 (42.5)	23 (57.5%)

Total	470	387	83

Notes: NOS = not otherwise specified.

**Table 3:** Univariate analysis to determine distinguishing features of final malignancy and benign diagnoses of 308 nondiagnostic biopsies.

Characteristics	Malignancy (*n* = 78)	Benign (*n* = 230)	*P*
Age (years)	56.1 ± 10.9	54.7 ± 13.2	0.372^a^
Gender			0.708^b^
Male	58	166	
Female	20	64	
Lesion location 1			<0.001^b^
Left lung	33	103	
Right lung	45	127	
Lesion location 2			<0.001^b^
Upper lobe	36	75	
Middle and lower lob	42	155	
Lesion size (mm)	38.8 ± 21.4	29.9 ± 14.4	0.001^b^
≤15 mm	7	25	
>15 mm	71	205	
Lesion type			0.087^b^
Solid	63	163	
Subsolid	15	67	
Patient position			0.01^b^
Supine	18	33	
Lateral	16	88	
Prone	44	109	
Needle type (gage)			0.874^b^
16	6	19	
18	72	211	
Puncture angle			0.612^c^
0°	20	50	
15°	49	160	
30°	3	9	
Multiangle	6	11	
No. of tissue samplings			0.577^b^
≤2	22	77	
3	41	118	
>3	15	35	
Nondiagnostic			0.001^c^
Nonspecific benign	45	212	
Atypical cell present	10	1	
Insufficient	23	17	

*Notes*. ^a^one-way analysis of variance. ^b^Pearson Chi-square. ^C^Fisher exact test.

**Table 4:** Results of multivariate logistic regression analysis to determine distinguishing features of final malignant and benign diagnoses of nonmalignant biopsies.

Variables/categories	*B*	SD	*P*	OR	95% CI
Lesion size (mm)	0.024	0.009	0.005	1.025	1.007–1.043
Lesion location 1 (ref. = left lung)					
Right lung	0.138	0.309	0.657	1.147	0.626–2.104
Lesion location 2 (ref. = upper lobe)					
Middle and lower	0.569	0.351	0.105	1.766	0.887–3.516
Patient position (ref. = supine)					
Lateral	−0.699	0.464	1.132	0.497	0.20–1.234
Prone	0.147	0.445	0.742	1.158	0.484–2.771
Lesion type (ref. = solid)					
Subsolid	0.803	0.381	0.035	2.321	1.057–4.712
Nondiagnostic (ref. = non-specific benign)					
Atypical cell present	3.539	1.103	0.001	34.421	3.959–299.263
Insufficient	1.922	0.386	<0.001	6.837	3.210–14.563

**Table 5:** Nonrepeated biopsies with final malignant diagnosis and methods by which their true diagnoses were determined (*n* = 58).

Malignancy (*n* = 58)	TBLB	TBNA	Lymph node (biopsy)	Smear cell and cell block	Open thoracotomy	Follow-up
Small cell lung cancer	5	2	1			
Squamous cell carcinoma	19	1	1			
Adenocarcinoma	7	2	1	2	3	
Pulmonary mucoepidermoid carcinoma	1					
Lung adenosquamous carcinoma		1				
Lymphoepithelioma-like carcinoma		1				
Pulmonary sequestration	1				2	
Mixed epithelial-mesenchymal tumor					1	
Metastasis						
Clinically malignancy						9

## Data Availability

The data used to support the findings of this study are available from the corresponding author upon request.
